# Study of Risk Factors for Total Attack Rate and Transmission Dynamics of Norovirus Outbreaks, Jiangsu Province, China, From 2012 to 2018

**DOI:** 10.3389/fmed.2021.786096

**Published:** 2022-01-07

**Authors:** Jing Ai, Yuanzhao Zhu, Jianguang Fu, Xiaoqing Cheng, Xuefeng Zhang, Hong Ji, Wendong Liu, Jia Rui, Jingwen Xu, Tianlong Yang, Yao Wang, Xingchun Liu, Meng Yang, Shengnan Lin, Xiaohao Guo, Changjun Bao, Qun Li, Tianmu Chen

**Affiliations:** ^1^Jiangsu Provincial Center for Disease Control and Prevention, Nanjing, China; ^2^Key Laboratory of Enteric Pathogenic Microbiology, Ministry of Health, Jiangsu Provincial Center for Disease Control and Prevention, Nanjing, China; ^3^State Key Laboratory of Molecular Vaccinology and Molecular Diagnostics, School of Public Health, Xiamen University, Xiamen, China; ^4^Public Health Emergency Center, Chinese Center for Disease Control and Prevention, Beijing, China

**Keywords:** infectious diseases, norovirus, total attack rate, transmission dynamics, risk factors

## Abstract

**Objective:** To describe the epidemiological characteristics of norovirus outbreaks in Jiangsu Province, utilize the total attack rate (*TAR*) and transmissibility (*R*_unc_) as the measurement indicators of the outbreak, and a statistical difference in risk factors associated with *TAR* and transmissibility was compared. Ultimately, this study aimed to provide scientific suggestions to develop the most appropriate prevention and control measures.

**Method:** We collected epidemiological data from investigation reports of all norovirus outbreaks in Jiangsu Province from 2012 to 2018 and performed epidemiological descriptions, sequenced the genes of the positive specimens collected that were eligible for sequencing, created a database and calculated the *TAR*, constructed SEIAR and SEIARW transmission dynamic models to calculate *R*_unc_, and performed statistical analyses of risk factors associated with the *TAR* and *R*_unc_.

**Results:** We collected a total of 206 reported outbreaks, of which 145 could be used to calculate transmissibility. The mean *TAR* in was 2.6% and the mean *R*_unc_ was 12.2. The epidemiological characteristics of norovirus outbreaks showed an overall increasing trend in the number of norovirus outbreaks from 2012 to 2018; more outbreaks in southern Jiangsu than northern Jiangsu; more outbreaks in urban areas than in rural areas; outbreaks occurred mostly in autumn and winter. Most of the sites where outbreaks occurred were schools, especially primary schools. Interpersonal transmission accounted for the majority. Analysis of the genotypes of noroviruses revealed that the major genotypes of the viruses changed every 3 years, with the GII.2 [P16] type of norovirus dominating from 2016 to 2018. Statistical analysis of *TAR* associated with risk factors found statistical differences in all risk factors, including time (year, month, season), location (geographic location, type of settlement, type of premises), population (total number of susceptible people at the outbreak site), transmission route, and genotype (*P* < 0.05). Statistical analysis of transmissibility associated with risk factors revealed that only transmissibility was statistically different between sites.

**Conclusions:** The number of norovirus outbreaks in Jiangsu Province continues to increase during the follow-up period. Our findings highlight the impact of different factors on norovirus outbreaks and identify the key points of prevention and control in Jiangsu Province.

## Introduction

Norovirus, initially known as winter vomiting disease, was identified as the cause of the outbreak in 1972 when researchers observed 27-nm-virus particles by immune electron microscopy in the infected fecal filtrate from an acute outbreak of gastroenteritis in Norwalk, Ohio, in 1968, and named “Norwalk virus” (1, 2). Norovirus is a single-stranded and unenveloped RNA virus, which can be classified into 10 genogroups (GI-GX) and 49 genotypes with most of the infections in humans caused by GI and GII genogroups, which could be further divided to 9 and 27 genotypes, respectively (3). Currently, no drugs effectively treat norovirus.

The disease burden of norovirus is becoming increasingly serious worldwide and in China, causing ~684 million cases and 212,000 deaths, annually (4). Compared with other countries, China has a higher prevalence of norovirus (19.8–21.0%) (5). The annual report of the National Statutory Infectious Diseases Reporting System, found a significant decrease in the incidence of infectious diarrhea caused by cholera, dysentery and typhoid fever between 2006 and 2016; on the other hand, the number of cases caused by viral pathogens increased from 741,809 to 1,017,962 (6). Nowadays, norovirus is replacing rotavirus as the main pathogen of viral acute gastroenteritis, which indicates that norovirus outbreaks remain an important public health problem in China. Understanding the characteristics of the disease is key to the effective implementation of disease prevention and control. Existing studies have found significant differences in the epidemiological characteristics of norovirus outbreaks between China and other countries. First, whole populations are susceptible to norovirus infection (7); however, the outbreak sites are different. In developed countries, norovirus outbreaks occur mainly in medical institutions, such as long-term care facilities and hospitals (8, 9), whereas in China, norovirus outbreaks mainly occur in school environments and nurseries (10–13). Second, different age groups face different risks of infection; particularly, one study found that the highest incidence was observed in children aged <5 years (14). Other studies have found that children younger than 12 years are more susceptible and infection is common in children under 2 years old in developing countries (15, 16). Additionally, differences were observed between the genotypes. In the past two decades, the GII.4 virus has caused norovirus outbreaks in most age groups worldwide (17). However, norovirus genotypes are constantly changing in China with the emergence of new genotypes and differences in genotypes observed in different regions.

In recent years, an increase has been observed in the incidence rate of infectious diarrhea in Jiangsu Province. In a study on norovirus and meteorological factors in Jiangsu Province, bacterial culture and viral nucleic acid testing were performed on 6,640 stool specimens collected, and 1,193 positive specimens were obtained, of which the positive rate for viruses was greater than that for bacteria; and among the positive specimens for viruses, the positive rate for norovirus was higher than that for rotavirus (18). Norovirus has a heavy disease burden in Jiangsu Province. Currently, researchers in Jiangsu Province have made significant progress in studying the molecular epidemiological characteristics of norovirus outbreaks (19–22). However, epidemiological characterization of large sample sizes is still limited for a large number of reported norovirus outbreaks in Jiangsu Province. Previous epidemiological studies on norovirus outbreaks usually calculated the total attack rate (*TAR*) or duration of the outbreak as the dependent variable and collected risk factors associated with the infection as independent variables for statistical analysis, and finally found the epidemiological characteristics of norovirus outbreaks and factors influencing the severity of outbreaks in a certain area during a certain period of time, providing a scientific basis for controlling norovirus outbreaks (23–25). However, we believe that studies on the *TAR* do not completely reflect the transmissibility of the virus, and it is necessary to construct mathematical models to quantify the transmissibility of norovirus in outbreaks. Current mathematical models used to calculate the transmissibility of infectious diseases include agent-based models and ordinary differential equation models. In our previous study, the transmissibility of norovirus in interpersonal transmission and water transmission modes was described by constructing ordinary differential equation models (26). Therefore, in order to identify as many risk factors as possible and determine the epidemiological characteristics of norovirus outbreaks in Jiangsu province, we collected all outbreak investigation reports from 2012 to 2018 in Jiangsu Province, sequenced all positive specimens eligible for sequencing to determine the genotype, determined the *TAR* of each outbreak, and constructed transmission dynamics models for different transmission routes to calculate the transmissibility, and performed statistical analyses of risk factors associated with the *TAR* and transmissibility.

## Materials and Methods

### Research Design

**Figure 1:**
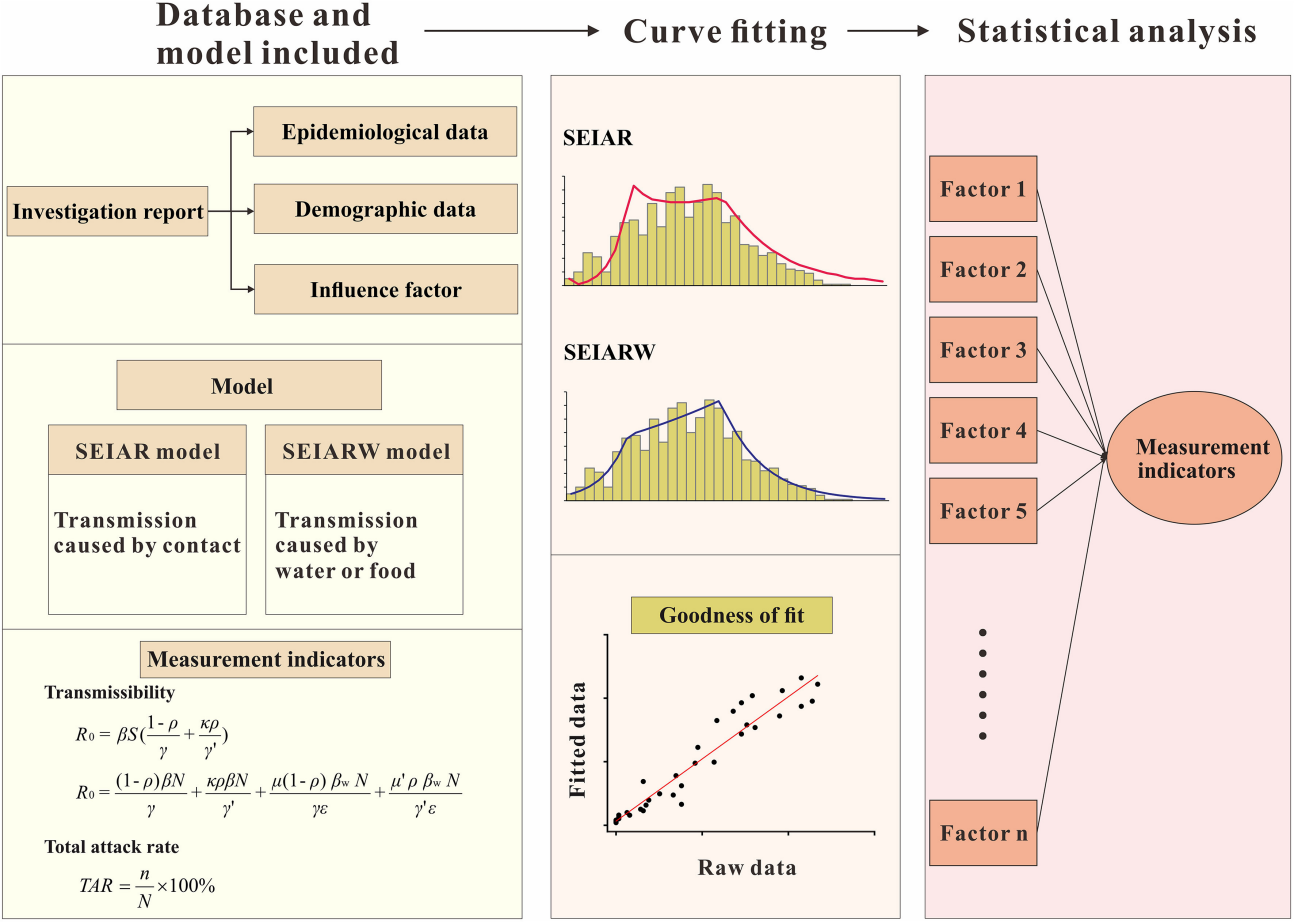
Flow chart of norovirus outbreak research in Jiangsu Province.

Our study is an observational study that combines fieldwork and transmissibility. This study consists of three parts, the logical relationship of which is shown in Figure 1. According to the investigation report, we established a database, numbered each outbreak, and transformed the description of the outbreak into tabular data. We could find out epidemiological data, demographic data and influencing factors from investigation reports. In order to identify genotypes, we also collected and detected samples. From the report, we could know exactly the transmission route of each outbreak, and select different transmission models according to different transmission routes. The independent variable was the data extracted from each investigation report; the dependent variables were the *TAR* and reproduction number (*R*_0_). The *TAR* was calculated according to the outbreak data, and *R*_0_ was calculated using the transmission dynamics model. In this study, prevalence was used for model fitting. The transmission dynamics model aims to refine the transmission process of infectious diseases in the whole population, so as to explain the transmission mechanism of infectious diseases. According to the influencing factors in the report, we explored whether each influencing factor would affect the *TAR* and *R*_0_. Chi-square analysis was used to compare the rates of multiple samples, and analysis of variance was used to compare the mean of multiple samples.

### Data Sources

#### Database

The outbreak database (Supplementary Table 1) contained the city where the outbreak occurred, the number of people affected by the outbreak (susceptible people), the number of cases, the number of deaths, the location type, the onset date of the initial case, the transmission route and genotype. The data source of the outbreak database was mainly based on investigation reports. Contents of the report mainly included the investigation process and contents of the aggregated epidemic and outbreak, mainly including the basic situation investigation of the epidemic occurrence institution, on-site epidemiological investigation, hygienic investigation and epidemic termination evaluation, as well as the sample collection and laboratory test results involved in the investigation. All contents of the investigation report were specified in the “Guidelines on outbreak investigation, prevention and control of norovirus infection” issued by China Center for Disease Control and Prevention (27).

Case definition:

(1) Suspected case: That is, acute gastroenteritis cases, defined as those who defecate ≥ 3 times within 24 h and have character changes (dilute watery stool), and/or vomit ≥ 2 times within 24 h. (2) Clinical diagnostic cases: In the cluster epidemic or outbreak caused by norovirus infection, the cases that meet the definition of suspected cases and are epidemiologically related to laboratory diagnosed cases. (3) Laboratory diagnosed cases: In suspected cases or clinical diagnostic cases, stool, anal swab or vomit samples are positive for norovirus nucleic acid or ELISA antigen.

Judgment criteria of cluster epidemic and outbreak:

(2) Cluster epidemic: Within 3 days, 5 or more epidemiologically linked cases of norovirus infection occur in the same school, childcare institution, medical institution, nursing home, factory, construction site, cruise ship, community or village, and other crowded places or sites, of which at least 2 are laboratory diagnosed cases. (2) Outbreak: 20 or more epidemiologically linked cases of norovirus infection in the same school, childcare institution, medical institution, nursing home, factory, construction site, cruise ship, community or village, and other crowded places or sites within 7 days, of which at least 2 are laboratory diagnosed cases. (3) If no laboratory testing capability for norovirus is available or in the early stage of outbreak detection, a suspected outbreak or outbreak of norovirus infection can be determined if the following four characteristics of Kaplan Criteria are met: (1) more than half of the patients present vomiting symptoms; (2) average incubation period of 24–48 h; (3) average duration of illness 12–60 h; (4) exclude bacterial, parasitic and other pathogenic infections. The sensitivity of the Kaplan Criteria for identifying norovirus outbreaks is 68% and the specificity is 99%.

#### Laboratory Detection

All stool specimens collected from outbreaks would be tested for norovirus. The stool suspensions were diluted 10-fold with saline solution. Viral nucleic acids were extracted and tested using the MagMAXTM-96 Viral RNA Isolation Kit (Applied Biosystems, Foster City, CA) and the Qiagen Probe reverse-transcriptase polymerase chain reaction (RT-PCR) Kit (Qiagen, Hilden, Germany) on a 7500 real-time PCR platform (Applied Biosystems, Foster City, CA) with primers as described (28).

#### Genotyping and Phylogenetic Analysis

In our study, norovirus nucleic acid was detected in samples collected from each outbreak. All norovirus-positive samples were detected with a region of 543 bp (GI) or 557 bp (GII) targeted at the ORF1/ORF2 junction of the viral genome by one-step RT-PCR for norovirus genotyping (29). If sequencing failed, the VP1 gene in ORF2 was amplified using the previously described primers G1SKF/G1SKR(GI) or G2SKF/G2SKR(GII) (30). However, the CT values of some samples were >33, the viral load was low, and sufficient amplification products could not be obtained, meaning that sequencing conditions were not met and sequencing failed. The genotypes were determined using the norovirus automated genotyping tool (http://www.rivm.nl/mpf/norovirus/typing tool) and the human calicivirus typing tool (http://norovirus.ng.philab.cdc.gov). We selected 25 representative gene sequences for gene polymorphism demonstration, which covered all genotypes of norovirus causing norovirus outbreaks in Jiangsu Province from 2012 to 2018. Phylogenetic trees were constructed using the maximum likelihood algorithm with 1,000 bootstrap replicates and a Kimura2-parameter model in MEGA 7.0 (31); the norovirus reference sequences were obtained from the GenBank database. Nucleotide sequences obtained from this study were deposited in GenBank under the accession numbers MZ373204-MZ373210 and MZ373215-MZ373233.

### Transmission Model

Similar to a previous study (32), a susceptible-exposed-symptomatic-asymptomatic-recovered (SEIAR) and a susceptible-exposed-symptomatic-asymptomatic-recovered-transmission media (SEIARW) model was employed for the simulation. The SEIAR model of interpersonal transmission and SEIARW model of water or food transmission were used to assess the transmissibility of norovirus in each outbreak. In this study, Berkeley Madonna 8.3.18 (developed by Robert Macey and George Oster of the University of California at Berkeley) was used for curve fitting and simulation, and SPSS 21.0 (IBM Company, U.S.A.) was used to calculate the coefficient of determination (*R*^2^) and *P*-value.

#### Interpersonal Transmission

In the SEIAR model, the population was divided into five groups: susceptible (*S*), exposed (*E*), symptomatic (*I*), asymptomatic (*A*), and recovered (*R*)

**Figure 2:**
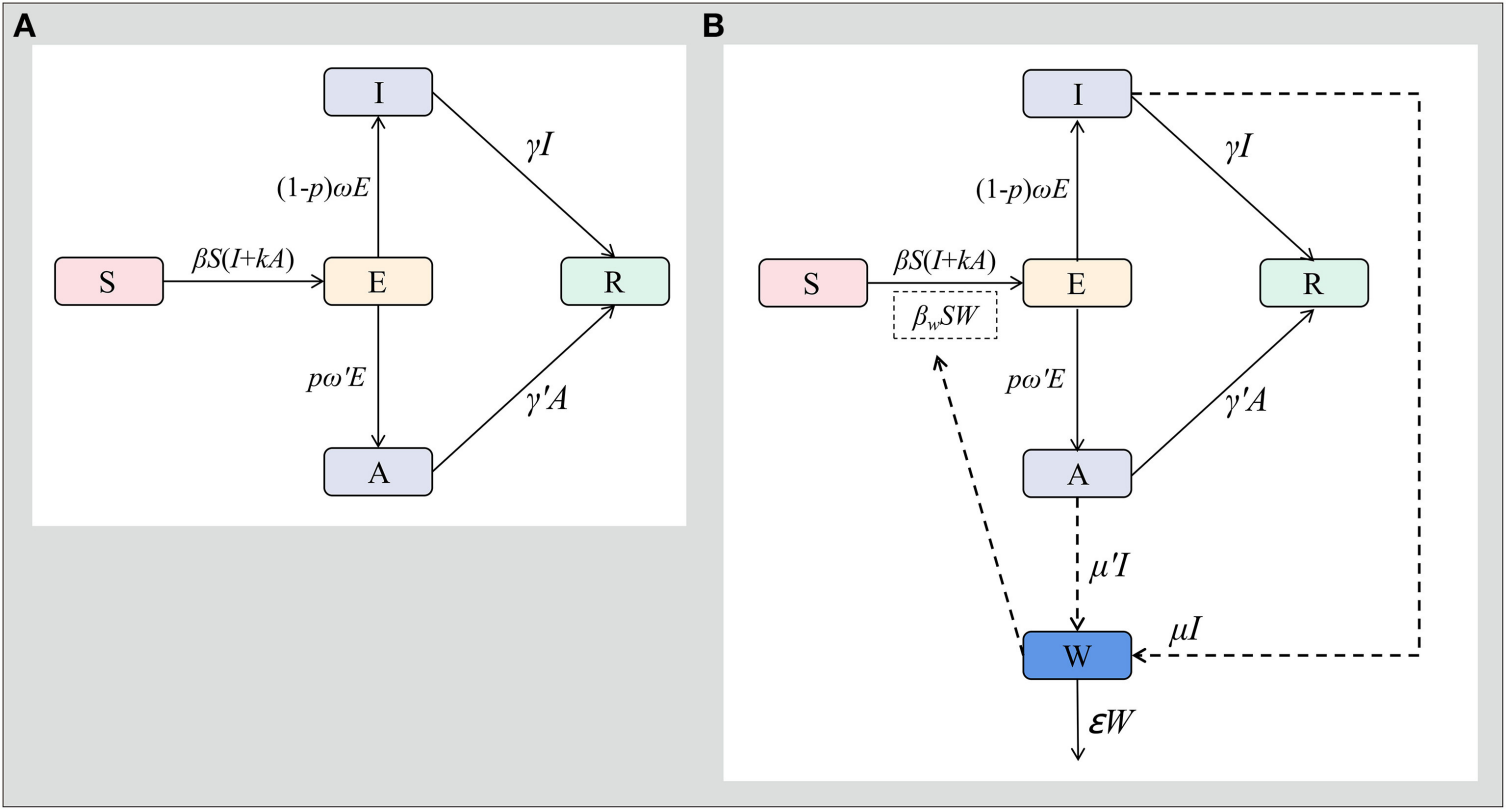
SEIAR model and SEIARW model for norovirus transmission in Jiangsu Province. [**A**: susceptible-exposed-symptomatic-asymptomatic-recovered model (SEIAR model), **B**: susceptible-exposed-symptomatic-asymptomatic-recovered-transmission media model (SEIARW model)].

The assumptions of the model are as follows:
A susceptible person becomes the exposed person after coming into contact with the symptomatic and asymptomatic infected person at the speed of β*SI* and β*S*κ*A*. Where β represents the probability of transmission per contact, and κ represents the transmissibility of asymptomatic cases compared to symptomatic cases.
After the incubation period (1/ω) and latent period (1/ω′), the exposed persons become symptomatic or asymptomatic; where ρ represents the proportion of asymptomatic infections.
Symptomatic and asymptomatic cases recovered after a period of 1/γ and 1/γ′.
The flowchart of the model is shown in Figure 2A.

The equations of the model are as follow:


$$\begin{array}{lll} \frac{dS}{dt} & = & -\beta S\left(I+\kappa A\right)\\ \frac{dE}{dt} & = & \text{β}S\left(I+\kappa A\right)-\left(1-\rho \right)\omega E-\rho \omega^{\prime }E\\ \frac{dI}{dt} & = & \left(1-\rho \right)\omega E-\gamma I\\ \frac{dA}{dt} & = & \rho \omega^{\prime }E-\gamma^{\prime }A\\ \frac{dR}{dt} & = & \gamma I+\gamma^{\prime }A \end{array}$$

#### Water or Food Transmission

In the SEIARW model, the water or food transmission medium (*W*) is added.

The assumptions of the model are as follows:

Both symptomatic and asymptomatic cases contaminate water or food by shedding pathogens into compartment *W* at shedding rates of μ*I* and μ'*A*. μ and μ' represent shedding coefficients.

When exposed to contaminated water or food, the susceptible people will become exposed people at the rate of β_w_*SW*, which represents the coefficient of water or food transmission.

The flowchart of the model is shown in Figure 2B.

The equations of the model are as follow:


$$\begin{array}{lll} \frac{dS}{dt} & = & -\beta S\left(I+\kappa A\right)-\beta_{w}SW\\ \frac{dE}{dt} & = & \text{β}S\left(I+\kappa A\right)+\beta_{w}SW-\left(1-\rho \right)\omega E-\rho \omega^{\prime }E\\ \frac{dI}{dt} & = & \left(1-\rho \right)\omega E-\gamma I\\ \frac{dA}{dt} & = & \rho \omega^{\prime }E-\gamma^{\prime }A\\ \frac{dR}{dt} & = & \text{γ}I+\gamma^{\prime }A\\ \frac{dW}{dt} & = & \varepsilon \left(I+ca-w\right) \end{array}$$

#### Parameter Source

Model 1 included seven parameters: β*, k*, ω, ω′, ρ, γ and γ′ (Table 1). β was adjusted using the actual outbreak data and model. Previous researches have shown that the incubation period of norovirus was 12–48 h (33, 34), therefore, our model chose 1 d as the incubation period (ω = 1). A study showed that norovirus started to shed within an average of 36 h (range: 18–110) after infection, and the virus could be discharged for approximately 26 d (range: 11–54) (35), thus, the latent and infectious period was 1 *d* and 26 *d*, so ω′=1, γ′ = 0.03846. The course of norovirus infectious diarrhea is generally 1–5 d (36, 37), with some cases reaching 4–6 d (38, 39). According to the Kaplan principle of norovirus infectious diarrhea diagnosis commonly used in the United States (40), the average course of the disease is 1–3 d. In this study, 3 d were used for the modeling study, namely γ = 0.3333. The asymptomatic infection ratio of this disease potentially reaches 30% (35, 41, 42); therefore, ρ = 0.3 was used.

**Table 1:** Parameter definitions and values.

**Parameter**	**Description**	**Unit**	**Value**	**Method**
β	Person–to-person contact rate	km^2^·individuals^−1^·day^−1^	–	Curve fitting
β_w_	Reservoir– to-person contact rate	mL^3^·cells^−1^·day^−1^	–	Curve fitting
*K*	Relative transmissibility rate of asymptomatic to symptomatic individuals	1	–	Curve fitting
Ω	Relative incubation rate*	day^−1^	1	(33, 34)
*ω′*	Relative latency rate*	day^−1^	1	(35)
*P*	Asymptomatic infection ratio	1	0.3	(35, 41, 42)
Γ	Recovery rate of the infected	day^−1^	0.3333	(36–40)
*γ′*	Recovery rate of the asymptomatic	day^−1^	0.03846	(35)
*?*	Pathogen lifetime relative rate	day^−1^	0.1	(44–52)
*?*	Person–to-reservoir contact rate (“shedding” by Infectious)	cells·mL^−3^·day^−1^·km^2^·individuals^−1^	–	c/*μ′* (43)
*μ′*	Person–to-reservoir contact rate (“shedding” by Asymptomatic)	cells·mL^−3^·day^−1^·km^2^·individuals-1	–	c/μ (43)
*C*	Shedding rate of the asymptomatic comparing to the infectious	1	–	Curve fitting

* *Incubation period is the time elapsed between infected and symptoms are first apparent, and latent period means the time from infected to infectiousness, in this table, incubation period = 1/ω, latent period = 1/ω′*.

Model 2 included 14 parameters. The normalized 10 parameters were the parameters that required specific values, which were *b, b*_*w*_, κ, ω, ω′, ρ, γ, γ′*, c*, and ε. Among them, *b* and *b*_*w*_were fitted using the actual outbreak data and model.

The shedding rate of the asymptomatic compared to the infectious was *c*, and μ′ = *c/*μ (43); subsequently, the specific value of the parameter was calculated by fitting the actual epidemic data. The survival time of norovirus in the external environment was approximately about 7–12 d (44–46), and the longest was 21–28 d (47–52). In this study, 10 d were used for the study; therefore, ε = 0.1 was used.

### Evaluation Index

In this study, *R*_0_ was used to evaluate the transmissibility of norovirus. In Model 1, the *R*_0_ formula is expressed as follows:


$$\begin{array}{l} R_{0}=\text{β}S\left(\frac{1-\rho }{\gamma }+\frac{\kappa \text{ρ}}{\gamma^{\prime }}\right) \end{array}$$

In Model 2, *R*_0_ formula is expressed as follows:


$$\begin{array}{l} R_{0}=\frac{\left(1-\rho \right)\text{β}N}{\gamma }+\frac{\text{κρβ}N}{\gamma^{{}^{\prime }}}+\frac{\mu \left(1-\rho \right)\beta_{w}N}{\gamma \varepsilon }+\frac{\mu^{{}^{\prime }}\rho \beta_{w}N}{\gamma^{\prime }\varepsilon } \end{array}$$

*R*_0_ was divided into two parts (*R*_unc_ and *R*_con_) where *R*_unc_ and *R*_con_ represent the uncontrolled and controlled *R*_0_, respectively. The epidemic curve was divided into two stages based on the increase and decrease. The increasing stage of the epidemic curve indicates that the intervention measures are yet to exert their effects; therefore, it is a better representation of the real transmissibility of the disease. Therefore, we used the data for epidemiological analysis and recorded the data as *R*_unc_.

*TAR* was used to evaluate the effects of various preventive and control measures. where *n* is the cumulative number of cases and *N* is the number of susceptible people in an outbreak.

The formula of *TAR* is:


$$\begin{array}{l} TAR=\frac{n}{N}\times 100\% \end{array}$$

### Statistical Method

All statistical analyses were performed using SPSS 21.0 (IBM Company, U.S.A.). For *TAR*, the chi-square test was used for comparison between the groups. Groups with significant differences were analyzed using the Bonferroni method for further pairwise comparisons. Statistical significance was set at *P* < 0.05 for both between group and pairwise comparisons. For *R*_0_, a homogeneity test of variance was first performed. If the variance was homogeneous, analysis of variance was used for inter-group comparison. Simultaneously, the LSD method was used for further pairwise comparison. If the homogeneity of variance was not satisfied, the Kruskal Wallis rank-sum test was used for inter-group comparison.

## Result

### Epidemiological Characteristics

**Figure 3:**
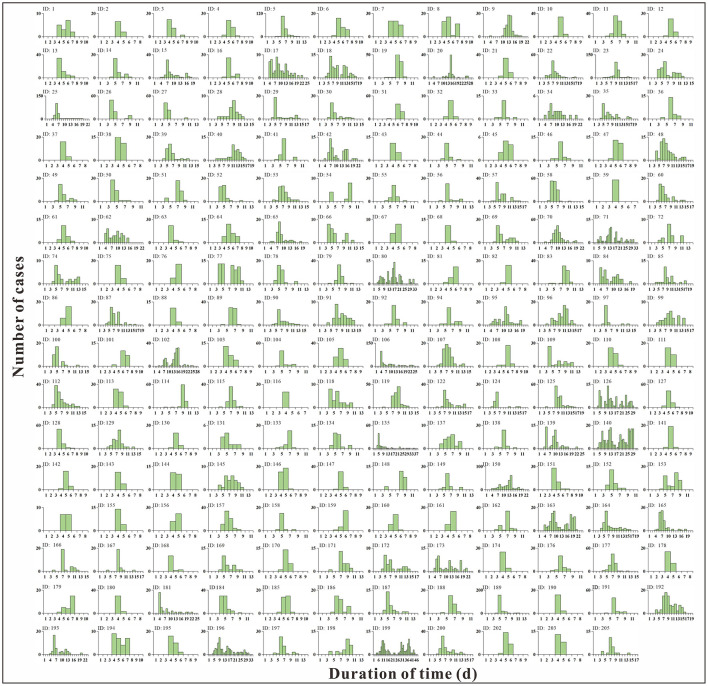
Epidemic curve of 206 outbreaks.

From 2012 to 2018, data from 206 norovirus outbreaks were collected in Jiangsu Province; all were able to calculate for the *TAR*. The epidemic curves of 206 outbreaks are showed in Figure 3. However, only 145 met the conditions for calculating transmissibility, and the basic reproduction number was calculated. The fitting effects of 145 outbreaks are shown in Supplementary Figure 1 which found the mean *R*^2^ was 0.76 and all *P*-values were significant.

For risk factors for *TAR* and transmissibility, we have the following findings (Tables 2, 3).

**Table 2:** TAR and transmissibility and influence factors in 206 outbreaks of norovirus in Jiangsu Province.

	**TAR (%)**			**R_***unc***_**		
	***N***	**Mean**	**95%CI**	***N***	**Mean**	**95%CI**
Influence factors	206	2.6		145	12.2	10.7–13.6
**Year**						
2012	4	1	0.9–1.2	3	11.3	1.2–21.4
2013	6	3.2	2.9–3.6	5	12.3	4.5–20.1
2014	15	1.6	1.5–1.7	9	10.4	5.9–15
2015	22	2	1.9–2.2	16	12.7	7.6–17.7
2016	14	1.5	1.4–1.6	11	11.9	7.3–16.4
2017	78	3.5	3.5–3.6	57	12.6	9.9–15.4
2018	67	2.5	2.4–2.6	44	11.9	9.5–14.3
**Month**						
1	8	2.2	1.9–2.4	5	8.1	0.4–15.8
2	27	3.7	3.5–3.9	20	17.9	11.7–24.2
3	37	3.7	3.5–3.8	32	10.4	7.2–13.5
4	17	3.1	2.9–3.3	11	12.6	7.9–17.4
5	5	1.5	1.3–1.6	4	7.8	4.4–11.2
6	6	1.4	1.1–1.6	2	10.4	0–39.7
7	0	NA	NA	0	NA	NA
8	0	NA	NA	0	NA	NA
9	7	2.4	2.2–2.6	5	7.1	1.7–12.5
10	25	2.9	2.7–3.1	15	12.4	9.2–15.6
11	39	2.4	2.3–2.5	25	12.8	9.5–16.2
12	35	1.8	1.7–1.9	26	11.6	8.6–14.6
**Seasons**						
Spring	59	3.2	3.1–3.3	47	10.7	8.3–13
Summer	6	1.4	1.1–1.6	2	10.4	0–39.7
Autumn	71	2.5	2.4–2.6	45	12.1	9.9–14.2
Winter	70	2.4	2.3–2.5	51	13.7	10.8–16.7
**City**						
Changzhou	46	3	2.9–3.1	37	10.5	8.2–12.8
Huai'an	3	1.4	1.1–1.7	3	10.5	0–26.2
Lianyungang	2	3.1	2.2–3.9	1	12.7	NA
Nanjing	20	2.4	2.3–2.5	15	17.3	12.0–22.5
Nantong	4	2.7	2.1–3.3	2	28.5	0–81.7
Suzhou	17	4.7	4.4–5	11	17.4	8.0–26.7
Taizhou	13	1.4	1.3–1.5	4	13.7	0–33.5
Wuxi	65	2.2	2.2–2.3	47	10.8	8.5–13
Suqian	3	0.4	0.3–0.5	3	8.1	1.9–14.3
Xuzhou	2	3.5	3.1–3.9	1	9.0	NA
Yancheng	6	1.9	1.7–2.1	5	8.6	0–18.4
Yangzhou	18	4.5	4.3–4.8	13	11.0	6.6–15.5
Zhenjiang	7	2.2	2–2.5	3	15.2	0–47.4
**Region**						
South of Jiangsu	155	2.7	2.6–2.7	113	12.3	10.6–14
Middle of Jiangsu	35	2.9	2.8–3.1	19	13.4	8.8–18.1
North of Jiangsu	16	1.7	1.6–1.8	13	9.3	5.9–12.6
**Rural and Urban**						
Rural	46	3.9	3.7–4	25	11.4	9.3–18.3
Urban	160	2.4	2.3–2.4	120	12.4	10.3–13.3
**Categories of places**						
Non-school places	10	8.4	7.5–9.3	8	7.6	2.9–12.3
Kindergarten	26	5.7	5.3–6.1	16	16.1	10.8–21.4
Primary school	108	2.4	2.3–2.5	73	13.5	11.4–15.5
Middle school	39	3.4	3.3–3.5	32	10.6	7.8–13.3
Common Colleges and Secondary vocational school	14	1.7	1.6–1.8	11	6.5	3.0–10
Nine-year school and Twelve-year school	9	1.7	1.5–1.9	5	10.9	0–28.9
**Population**						
0–999	61	5.6	5.3–5.8	39	13.4	10.3–16.5
1000–1999	70	3.3	3.2–3.4	53	12.8	10.4–15.1
2000–2999	45	2.6	2.5–2.7	35	11.2	8.2–14.1
≥3000	30	1.3	1.3–1.4	18	9.7	5.9–13.5
**Route of transmission**						
Water or food to person	8	2.2	2–2.3	7	14.0	5.3–22.7
Person to person	198	2.6	2.6–2.7	138	12.1	10.6–13.6
**Virus group**						
GI	9	1.3	1.1–1.4	7	10.3	4.1–16.6
GII	132	2.7	2.7–2.8	98	12.8	11.0–14.6
**Virus genotype**						
GII.2 [P16]	87	3.1	3–3.2	64	13.3	10.9–15.7
GII.17 [P17]	21	2.2	2–2.3	16	10.8	7.6–14.1
GII.3 [P12]	11	2	1.8–2.2	7	17.4	6.1–28.7
GII.4 [P31]	6	1.5	1.3–1.6	5	8.0	0–16.2
GI.6*	3	1.8	1.4–2.1	2	6.6	0–21.7
GII.6 [P7]	3	3.8	3.1–4.6	3	15.6	9–22.1
GI.2*	2	2.8	2.3–3.3	2	16.7	0–114.7
GI.2 [P2]	2	1.8	1.3–2.2	2	9.3	0–61.8
GI.3 [P13]	1	2.8	NA	NA	NA	NA
GI.6 [P11]	1	0.2	NA	1	7.2	NA
GII.1*	1	2.4	NA	1	14.5	NA
GII.13 [P16]	1	4.6	NA	1	3.4	NA
GII.14 [P7]	1	3.3	NA	1	4.5	NA
GII.2 [P2]	1	3.4	NA	NA	NA	NA

* *RdRp Genotype not detected*.

**Table 3:** Chi-square test and pairwise comparison of the differences in TAR of influencing factors.

**Classification**	**Variables**	**Cases**	**Non-case**	**χ^2^**	***P*** **-value**
Year	2012^a^	147	14,176	998.138	*P* < 0.05
	2013^b^	326	9,715		
	2014^c^	545	33,250		
	2015^d^	883	42,322		
	2016^c^	513	34,179		
	2017^b^	4,784	129,986		
	2018^e^	3,108	122,171		
Month	1^a,b^	391	17,783	925.107	*P* < 0.05
	2^c^	1,458	37,600		
	3^c^	2,268	59,199		
	4^d^	772	23,968		
	5^e^	263	17,663		
	6^e^	155	11,286		
	7				
	8				
	9^b^	494	20,324		
	10^d^	909	30,539		
	11^b^	1,961	79,623		
	12^a^	1,635	87,814		
Seasons	Spring^a^	3,303	100,830	237.62	*P* < 0.05
	Summer^b^	155	11,286		
	Autumn^c^	3,364	130,486		
	Winter^c^	3,484	143,197		
City	Changzhou^a^	2,679	87,170	1,458.876	*P* < 0.05
	Huai'an^b,c^	94	6,727		
	Lianyungang^a,d,e,f,g,h,i,j,k,l,m,n,o,p,q^	49	1,555		
	Nanjing^n,o,p,q^	1,080	44,012		
	Nantong^a,j,k,l,m,p,q^	80	2,844		
	Suzhou^i^	987	19,906		
	Taizhou^c^	414	29,444		
	Wuxi^g,h,l,m,o,q^	2,563	111,413		
	Suqian^r^	58	13,904		
	Xuzhou^a^	246	6,804		
	Yancheng^b,f,h,k,m^	334	16,926		
	Yangzhou^e,i^	1,334	28,114		
	Zhenjiang^d,f,g,h,j,k,l,m,n,o,p,q^	388	16,980		
Region	North of Jiangsu^a^	7,697	279,481	193.783	*P* < 0.05
	Middle of Jiangsu^b^	1,828	60,402		
	South of Jiangsu^c^	781	45,916		
Rural and Urban	Rural^a^	2,492	61,506	502.826	*P* < 0.05
	Urban^b^	7,814	324,293		
Categories of places	Non-school places^a^	320	3,477	1,565.869	*P* < 0.05
	Kindergarten^b^	767	12,661		
	Primary school^c^	4,958	202,153		
	Middle school^d^	2,706	76,743		
	Common Colleges and Secondary vocational school^e^	1,112	65,204		
	9-year school and 12-year school^e^	443	25,561		
Population	0–999^a^	1,929	32,570	22.19	*P* < 0.05
	1,000–1,999^b^	3,580	106,354		
	2,000–2,999^c^	2,890	106,526		
	≥3,000^d^	1,907	140,349		
Route of transmission	Water or food to person^b^	627	28,182	2,296.419	*P* < 0.05
	Person to person^a^	9,679	357,617		
Virus group	GI^a^	353	27,758	220.681	*P* < 0.05
	GII^b^	6,921	245,709		
Virus genotype	GII.2 [P16]^a^	5,082	160,556	677.818	*P* < 0.05
	GII.17 [P17]^b,c,d,e^	835	37,256		
	GII.3 [P12]^d,e^	402	20,094		
	GII.4 [P31]^f^	295	19,690		
	GI.6^c,e,f^	106	5,914		
	GII.6 [P7]^a,g^	99	2,487		
	GI.2^a,b^	133	4,579		
	GI.2 [P2]^b,c,d,e,f^	66	3,675		
	GI.3 [P13]^a,b,c,d,e,f,g^	18	620		
	GI.6 [P11]^h^	30	12,970		
	GII.1^a,b,c,d,e,f^	45	1,856		
	GII.13 [P16]^g^	117	2,438		
	GII.14 [P7]^a,b,c,d,e,g^	33	959		
	GII.2 [P2]^a,b,c,d,e,f,g^	13	373		

*The same letter of the mark indicates that there is no difference between the two groups of data, and the different letter indicates that the difference is statistically significant*.

*For example, the mean TAR in spring was statistically different from all other seasons; the mean TAR in summer was statistically different from all other seasons; the mean TAR in autumn was statistically different from spring and summer only, but not from winter; and the mean TAR in winter was statistically different from spring and summer only, but not from autumn*.

The mean *TAR* of 206 outbreaks was 2.6% and the mean *R*_unc_ of 145 outbreaks was 12.2 (95% confidence interval: 10.7–13.6) from 2012 to 2018.

**Figure 4:**
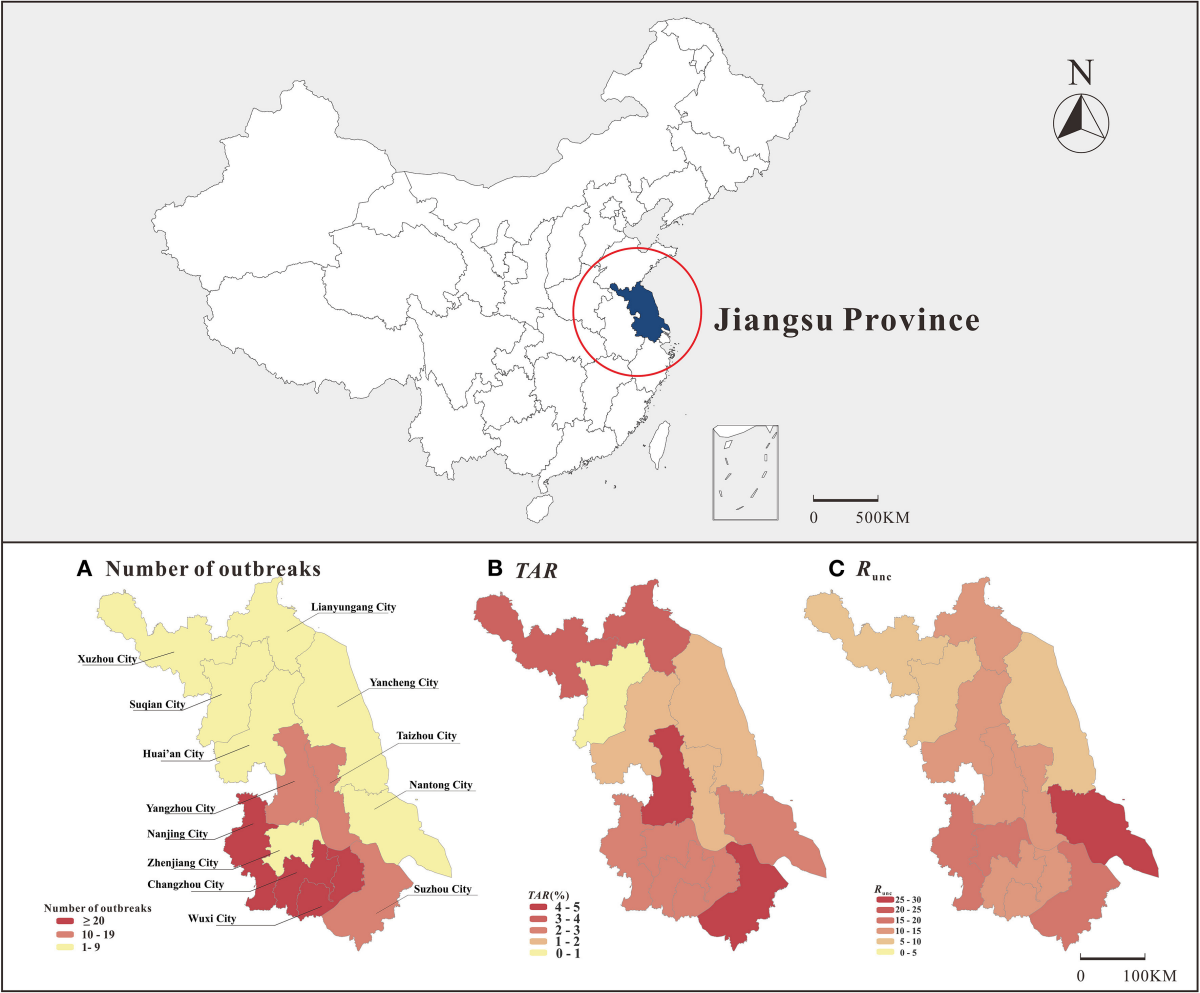
Distribution of number of outbreaks, *TAR* and transmissibility on map of Jiangsu Province (**A**: number of outbreaks, **B**: *TAR*, **C**: *R*_unc_).

The map showed significantly more outbreaks in southern Jiangsu compared to northern Jiangsu; furthermore, the *TAR* and *R*_unc_ showed the highest trend in southern Jiangsu (Figure 4). Wuxi City reported the most outbreaks (65 outbreaks), and Xuzhou City reported the least outbreaks (two outbreaks). There was a significant difference in mean *TAR* between cities (χ^2^ = 1,458.876, *P* < 0.05). The highest and lowest mean *TAR* were found in Suzhou City (4.7%) and Suqian City (0.4%), respectively. There was no significant difference in transmissibility between cities. Regarding location, the *TAR* between different cities was statistically different (χ^2^ = 193.783, *P* < 0.05), the highest and lowest mean *TAR* were observed in central and northern Jiangsu, respectively.

The number of outbreaks showed a significant upward trend from 2012 to 2018. A big turning point was observed in 2016–2017, with 14 and 78 outbreaks reported, respectively. There was a significant difference in mean *TAR* between the years analyzed (χ^2^ = 998.138, *P* < 0.05). Chi-square test pairwise comparison results showed differences between all years except 2013 and 2017, and 2014 and 2016. The mean *TAR* and *R*_unc_ tortuously changed over time, with a peak every 3 years.

In the four seasons, the number of outbreaks in summer was the lowest (six outbreaks); more outbreaks were observed in autumn and winter, 71 and 70, respectively. There was a significant difference in the mean *TAR* between seasons (χ^2^ = 237.62, *P* < 0.05). The summer had the lowest mean *TAR*. The mean *TAR* was found to be high from February to March and October (3.7, 3.7, 2.9%, respectively). The season significantly affected the *TAR* in all seasons except between autumn and winter. There was no significant difference in transmissibility between seasons. Outbreaks with high *R*_unc_ were concentrated in February, November, and April (17.9, 12.8, and 12.6, respectively).

Most outbreaks occurred in urban areas (160 outbreaks) compared to rural areas (46 outbreaks). The mean *TAR* between rural and urban areas was significantly different (χ^2^ = 502.826, *P* < 0.05). The mean *TAR* in rural areas was higher compared to urban areas (3.9 and 2.4%, respectively). The mean *R*_unc_ in rural areas and urban areas were 11.4 and 12.4, respectively.

Among 206 outbreaks, 196 occurred in schools, and ten occurred in places other than schools. There was a significant difference in the mean *TAR* between the categories of places analyzed (χ^2^ = 1,565.869, *P* < 0.05). Regarding the type of location, no significant difference was observed between common colleges and secondary vocational schools and 9-year school and 12-year school; however, other places significantly affected the mean *TAR*. The mean *TAR* in non-school places was 8.4%. Among the schools, kindergartens had the highest mean *TAR* (5.7%). There was no significant difference in transmissibility between the categories of places analyzed. Among the outbreak sites, the mean *R*_unc_ for kindergartens was 16.1, the mean *R*_unc_ observed in common colleges and secondary vocational schools was 6.5. Furthermore, the mean *R*_unc_ of non-schools was only 7.6.

Most of the outbreaks occurred in places with a small number of susceptible people, 61 and 70 outbreaks occurred in places with a number of <1,000 and 1,000–2,000 susceptible people, respectively, and the least outbreaks occurred in places with a small number of susceptible people, only 30. A comparison of the different numbers of susceptible people regarding the mean *TAR* showed statistically significant differences (χ^2^ = 22.19, *P* < 0.05). The highest and lowest *TAR* means were found in places with <1,000 people (5.6%) and places with more than 3,000 people (1.3%), respectively. Further, there was no significant difference in transmissibility between places with differing numbers of susceptible people, the mean *R*_unc_ of places with <1,000 people was 13.4, and the mean *R*_unc_ of places with more than 3,000 people was 9.7.

Regarding the transmission routes, 198 outbreaks were interpersonal transmission and eight were waterborne or foodborne transmission. The mean *TAR* was significantly different between different transmission routes (χ^2^ = 2,296.419, *P* < 0.05). The mean *TAR* of waterborne or foodborne transmission was lower compared to interpersonal transmission (2.2 and 2.6%, respectively). There was no significant difference in transmissibility between transmission routes. The mean *R*_unc_ of water or food transmission route and human transmission route was 14.0 and 12.1.

**Figure 5:**
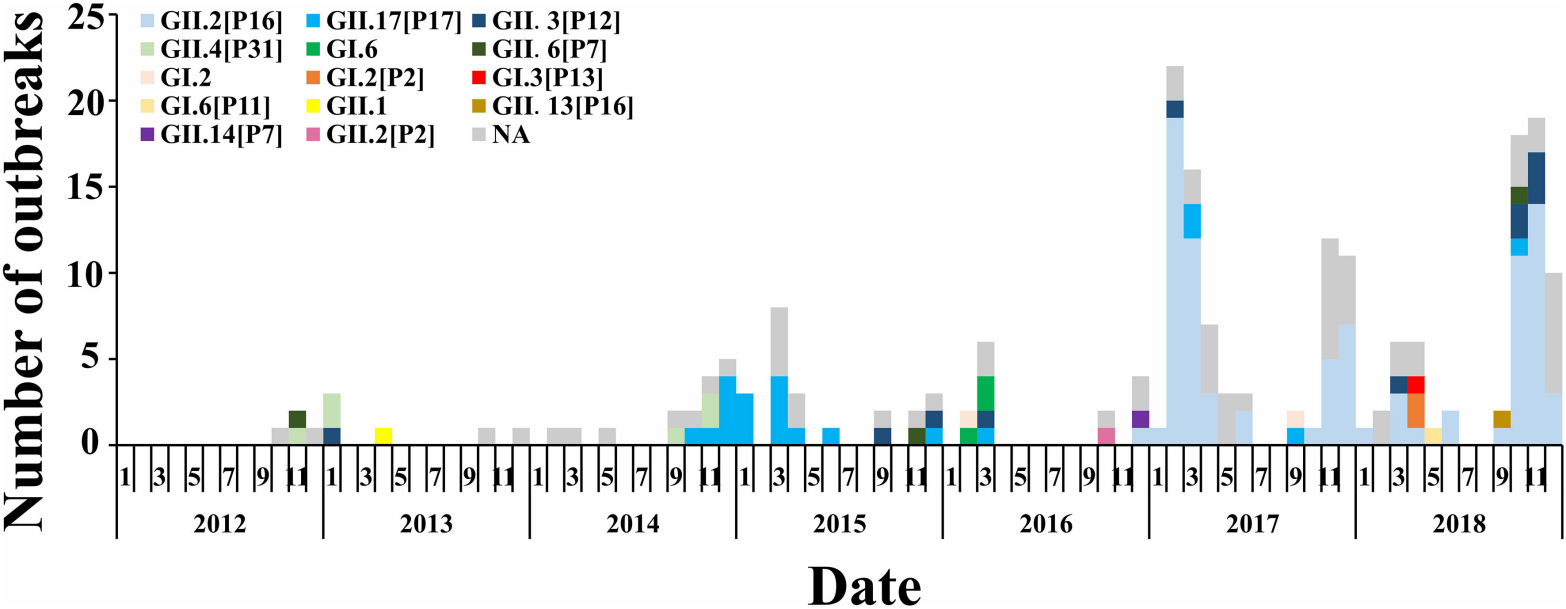
Changes of norovirus genotypes in Jiangsu Province from 2012 to 2018.

Genotype sequencing was completed in 141 outbreaks; among which, the GII genotype caused the highest number of outbreaks (132/141). Among the GII genotypes, the GII.2 [P16] genotype caused the most outbreaks (87 outbreaks). As shown in Figure 5, the outbreaks in 2012–2014 were mainly caused by the GII.4 [P31] and GII.P17 genotypes, and the majority of outbreaks in 2015–2016 were caused by the GII.17 [P17] genotype, while a change occurred after 2016 when the GII.2 [P16] genotype became dominant. In 2017 and 2018, outbreaks caused by the GII.2 [P16] genotype accounted for 91.0% (50/55) and 73.5% (36/49) of all outbreaks in that year, respectively. The GII.13 [P16] genotype had the highest mean *TAR* (4.6%), while GI.6 [P11] had the lowest (0.2%). Furthermore, the mean *R*_unc_ of GII.3 [P12] was 17.4, and the mean *R*_unc_ of GII.13 [P16] was 3.4.

### Genetic Diversity

To show the genetic diversity of norovirus in Jiangsu Province, 25 norovirus sequences with representative genotypes were analyzed using MEGA 7.0 software. Phylogenetic trees were constructed based on the partial RdRp gene (230 bp) and VP1 gene (280 bp) using the maximum likelihood method. As shown in Supplementary Figure 2, eight genotypes had discordant capsid and polymerase genotypes and were considered intergenotype recombinant strains (GI.6 [P11], GII.2 [P16], GII.3 [P12], GII.4 Sydney [P31], GII.6 [P7], GII.13 [P16], GI.3 [P13], and GII.14 [P7]), and three genotypes had accordant capsid and polymerase genotypes (GI.2 [P2], GII.2 [P2], and GII.17 [P17]).

## Discussion

In this study, we used *TAR* and a transmission dynamics model to explore which factors would influence the norovirus outbreaks in Jiangsu Province.

Regarding time, the number of reported cases increased sharply in 2017 and 2018. Jiangsu Province began to establish a norovirus surveillance system in 2012, using a unified case definition, outbreak determination principles, and laboratory testing methods for surveillance, so we consider our description that the number of norovirus outbreaks is increasing is consistent with the real epidemiologic characteristics. Meanwhile, we observed that the GII.2 [P16] genotype caused more than half of the norovirus outbreaks in Jiangsu province in 2017 and 2018. Previously, a study on norovirus outbreak surveillance in China had already found that the number of norovirus outbreaks increased substantially at the end of 2016, greatly exceeding the number reported in the same month in the previous 4 years, and the majority of these outbreaks were associated with GII.2 [P16] (53). The researchers analyzed this finding and concluded that the GII.2 [P16] genotype evolved extremely fast, which most likely led to a sudden increase of this virus (54). We, therefore, considered that the prevalence of the GII.2 [P16] genotype is responsible for the surge in the number of norovirus outbreaks in Jiangsu Province between 2017 and 2018.

Regarding the season, in our study, *TAR* was significantly lower during summer, and norovirus outbreaks in Jiangsu Province mainly occurred in the colder weather in spring, autumn, and winter, which is consistent with the results of previous studies (55, 56). Regarding the regional distribution, the increased outbreaks in southern Jiangsu despite the higher *TAR* in central Jiangsu may be attributed to the increased sensitivity to disease surveillance and more timely detection and reporting of the outbreaks in southern Jiangsu. Regarding rural and urban sites, the mean *TAR* in rural areas was higher compared to urban areas, which may be related to poor sanitation in rural areas and the untimely adoption of control measures.

Consistent with previous studies (8–13), the outbreaks we monitored occurred more often in schools than in hospitals and long-term care facilities, this phenomenon is largely due to the fact that: (1) In China (56), schools have a more standard outbreak screening system in which the government requires schools to check and screen students for symptoms of fever, vomiting, or diarrhea every morning. In contrast, hospitals or long-term care facilities do not see similar mechanisms for disease detection and reporting. (2) Long-term care facilities are important health care settings outside China. In the United States (57), long-term care facilities serve more than 2 million Americans each year, and local health units often provide infection control courses to facility staff. Ontario requires by law that each health unit must provide in-service training to long-term care facilities at least once a year. In France (58), epidemiological surveillance of acute gastroenteritis outbreaks in various types of nursing homes has been conducted since November 2010. Well-established facilities and systematic services make such settings capable of detecting and reporting norovirus outbreaks.

Most outbreaks occurred in primary schools, which is consistent with a previous study on norovirus outbreaks in China in 2014–2017 (13). Kindergarten has a higher *TAR* and transmissibility than other types of schools for the following reasons: (1) Studies have found that students in kindergartens and primary schools are more likely to vomit when infected with norovirus (59), and some children in kindergartens may become infected with the virus by going around when vomiting occurs due to poor hygiene awareness. (2) Kindergartens are small and therefore have a high population density and higher exposure among younger children. We found that the mean *TAR* of norovirus outbreaks was highest in outbreaks with <1,000 susceptible people, and of the 61 outbreaks reported in Jiangsu Province, each with <1,000 susceptible people, the highest proportion of outbreaks occurred in kindergartens, so it can be assumed that small crowded places such as kindergartens are more likely to cause the spread of the outbreak. (3) Different types of schools are composed of students of different ages. Since children under 5 years of age are most susceptible (60), and most of the students in kindergartens in China are children aged 3–6 years old, norovirus outbreaks are more likely to occur in kindergartens.

Jiangsu Provincial Center for Disease Control and Prevention has undertaken a national foodborne and waterborne norovirus outbreak surveillance program. Once an outbreak of norovirus occurs, the corresponding outbreak investigation will be started, the cases, water and food will be sampled, and the prevention and control measures will be implemented. However, due to the high economic level and satisfactory sanitary conditions in Jiangsu Province, norovirus outbreaks caused by water or food are rare, accounting for only eight (3.9%) of all reported outbreaks. Among them, water source outbreaks are caused by barreled water contamination rather than pipe network contamination, and foodborne outbreaks are caused by a small number of kitchen workers infecting and polluting the food, and not from the food itself. These conditions led to a small significant difference in *TAR* among outbreaks of different transmission routes.

The genetic diversity of noroviruses can be reflected by differences in epidemiology (61). Surveillance of norovirus genotypes in our study showed that the major genotypes of noroviruses in Jiangsu province change every 2–3 years. The following characteristics of the changes in the genotypes causing norovirus outbreaks have been noted: since 2002, GII.4 has been the predominant genotype in norovirus outbreaks in many countries. In the winter of 2014, the predominant norovirus strain in China changed to GII.17. Subsequently, in the winter of 2016 (2016–17), GII.2 [P16] became the predominant genotype causing norovirus outbreaks (56). Norovirus outbreaks caused by the GII.2 [P16] genotype were also found in other regions and countries outside mainland China, such as Hong Kong, Taiwan, Germany, Japan, France, the United States, and Australia. This genotype has also become the major genotype causing norovirus outbreaks in Hong Kong, Taiwan, and Germany (54). The changes in genotypes we observed in Jiangsu province are temporally consistent with the changes in genotypes throughout China. Epidemiological surveillance and vaccines are essential to control human pathogens (62). However, it has been found that different genotypes show different evolutionary patterns, and the production of new variants of GII.4 genotype every 2–3 years will always lead to a population that is always susceptible to GII.4 genotype of norovirus. Once an immune barrier is established, individuals will no longer be susceptible to the GII.2 virus, which is classified as a static virus (63, 64). Based on this view, our future studies intend to observe this through continuous monitoring of genotypes and changes in population incidence levels in combination with other factors.

The mean *R*_unc_ of norovirus outbreaks in Jiangsu Province was 12.2, which was close to the data from a previous study on outbreaks (2 to >14) (65). In our previous study, we used the model fitting method to adjust the value of κ (interpersonal transmission coefficient) to be closer to 0. Finally, we obtained transmissibility of 1.94 for norovirus outbreaks in the community, 3.44 for transmissibility in schools and 4.91 for transmissibility by water (26); these results were all smaller compared to the values obtained in the current study. This difference can be explained by another study in which researchers found that the transmissibility of norovirus was largely influenced by the structure of the model and the weight of asymptomatic infections (66). We, therefore, considered that differences in parameter values in the model and differences in sample size could be responsible for the higher transmissibility of norovirus outbreaks in Jiangsu Province.

According to our research, further improvements are required in the sensitivity monitoring in northern Jiangsu. According to the epidemiological characteristics of norovirus in Jiangsu Province, the surveillance of norovirus outbreaks should be strengthened in all seasons of the year. Furthermore, health education and health promotion should be accomplished before autumn and winter where the incidence of norovirus is high, especially in rural areas. For schools, kindergartens have the highest transmissibility, followed by primary schools. This phenomenon mainly considers the immunological differences between age groups and health habits. Older students who are not in kindergartens and primary schools, as well as teachers, should be more aware of norovirus and need to know how to handle vomit properly to prevent the spread of aerosols. Also, students who have gathered should be evacuated promptly and kept away from the vomit. Furthermore, suspected cases of norovirus infection should be isolated, especially kitchen staff. Finding out changes of dominant genotype has occurred is important for exploring the epidemiological characteristics of norovirus outbreaks in Jiangsu Province, however, not every positive specimen collected in an outbreak met the criteria for genetic sequencing and could be used to determine the genotype of the norovirus causing the outbreak. Therefore, tracking changes in the major genotypes causing outbreaks requires further surveillance.

## Conclusion

The number of norovirus outbreaks in Jiangsu Province has generally been on the rise during the follow-up period, so prevention and control remain an important issue. Currently, factors such as year, month, season, region, urban and rural type, place type, the population of outbreak sites, route of transmission, and virus genotype significantly impact the *TAR* of norovirus outbreaks; notably, different types of schools influence transmissibility. The dominant genotype of the virus will change every 3 years and surveillance of norovirus genotypes should be further strengthened to explore the transmissibility of different genotypes. Our study explored how these factors affect the *TAR* and transmissibility of norovirus outbreaks in Jiangsu Province, suggesting current priorities for the prevention and control of norovirus outbreaks in Jiangsu Province and future issues to be addressed in the surveillance of outbreaks.

## Data Availability Statement

The datasets presented in this study can be found in online repositories. The names of the repository/repositories and accession number(s) can be found in the article/Supplementary Material.

## Author Contributions

CB, QL, TC, JA, YZ, JF, and XC contributed to conception and design of the study. JA, JF, XC, XZ, HJ, and WL collected data. TC, QL, YZ, JA, JR, TY, YW, JX, XL, MY, SL, and XG analyzed the data. CB, TC, JA, YZ, JF, and XC wrote the first draft of the manuscript. All authors contributed to the article and approved the submitted version.

## Funding

This study was partly supported by the Key Medical Discipline of Epidemiology (No. ZDXK A2016008), the National Major S&T Projects (No. 2018ZX10714-002), the Jiangsu Provincial Medical Youth Talent (No. QNRC2016542), the Project of Jiangsu Commission of health (Z2019006), the NHC Key Laboratory of Echinococcosis Prevention and Control (No. 2020WZK2001), and project S202110384382 supported by XMU Training Program of Innovation and Entrepreneurship for Undergraduates.

## Conflict of Interest

The authors declare that the research was conducted in the absence of any commercial or financial relationships that could be construed as a potential conflict of interest.

## Publisher's Note

All claims expressed in this article are solely those of the authors and do not necessarily represent those of their affiliated organizations, or those of the publisher, the editors and the reviewers. Any product that may be evaluated in this article, or claim that may be made by its manufacturer, is not guaranteed or endorsed by the publisher.
