# Supplementary figures and images for: Study of Risk Factors for Total Attack Rate and Transmission Dynamics of Norovirus Outbreaks, Jiangsu Province, China, From 2012 to 2018
Source: Front Med (Lausanne). 2022 Jan 7;8:786096. doi: 10.3389/fmed.2021.786096 (PMC8777030; doi:10.3389/fmed.2021.786096)

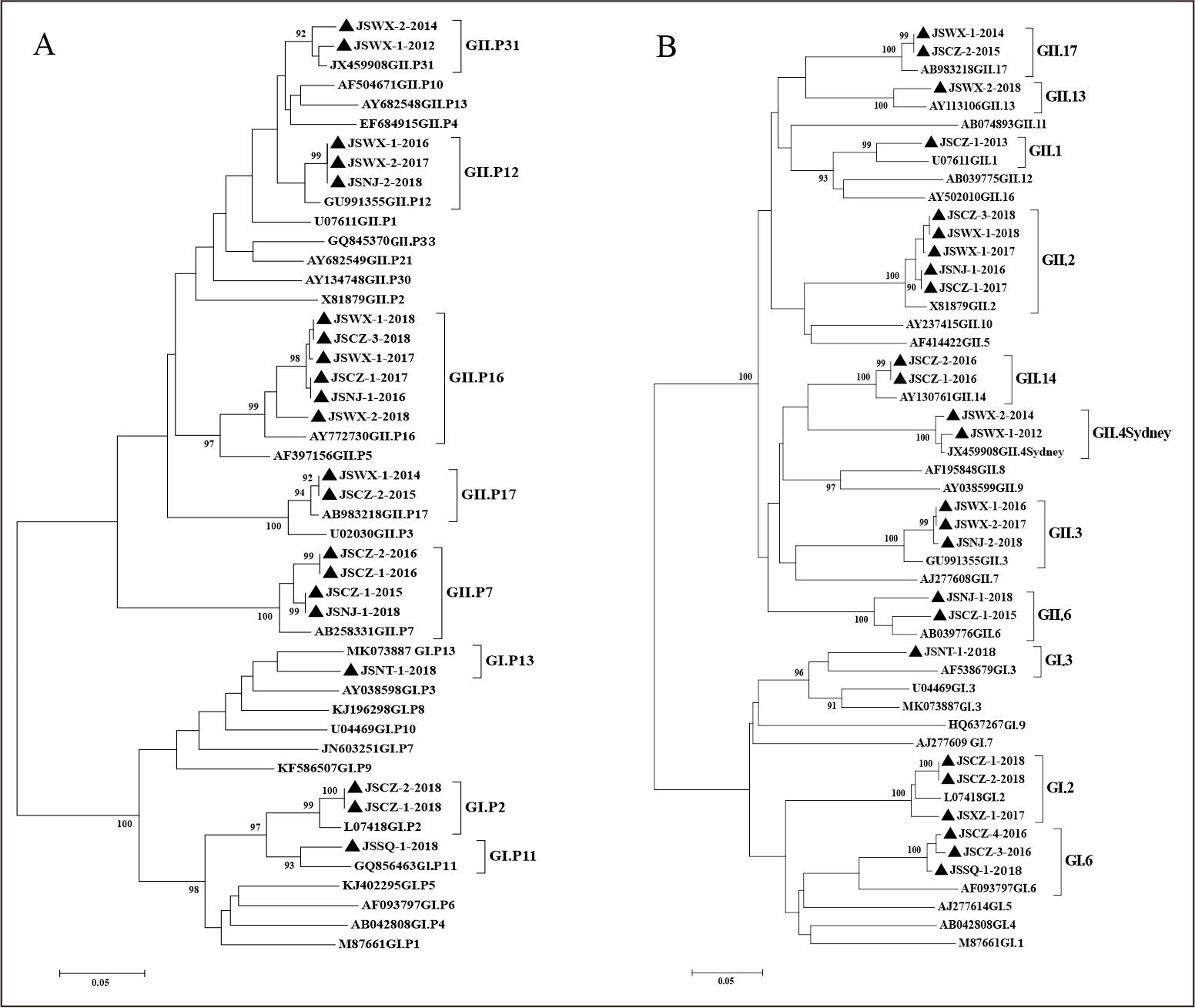

Supplement: Supplementary file 2 [file Image_2.JPEG]
